# Design and rationale of the inferior vena CAVA and Lung UltraSound-guided therapy in Acute Heart Failure (CAVAL US-AHF Study): a randomised controlled trial

**DOI:** 10.1136/openhrt-2022-002105

**Published:** 2022-11-07

**Authors:** Lucrecia Maria Burgos, Rocio Baro Vila, Ailin Goyeneche, Florencia Muñoz, Ana Spaccavento, Martin Andres Fasan, Franco Ballari, Martin Vivas, Laura Riznyk, Sebastian Ghibaudo, Marcelo Trivi, Ricardo Ronderos, Juan Pablo Costabel, Fernando Botto, Mirta Diez, CAVAL US-AHF group

**Affiliations:** 1 Heart Failure, Pulmonary Hypertension and Heart Transplant, Instituto Cardiovascular de Buenos Aires, Buenos Aires, Argentina; 2 Clinical Cardiology, Instituto Cardiovascular de Buenos Aires, Buenos Aires, Argentina; 3 Cardiac Imaging Department, Instituto Cardiovascular de Buenos Aires, Buenos Aires, Argentina; 4 Clinical Research, Instituto Cardiovascular de Buenos Aires, Buenos Aires, Argentina

**Keywords:** Diagnostic Imaging, Heart Failure, Heart Failure, Systolic, Heart Failure, Diastolic

## Abstract

**Background:**

Between 25% and 30% of patients hospitalised for acute heart failure (AHF) are readmitted within 90 days after discharge, mostly due to persistent congestion on discharge. However, as the optimal evaluation of decongestion is not clearly defined, it is necessary to implement new tools to identify subclinical congestion to guide treatment.

**Objective:**

To evaluate if inferior vena cava (IVC) and lung ultrasound (CAVAL US)-guided therapy for AHF patients reduces subclinical congestion at discharge.

**Methods:**

CAVAL US-AHF is a single-centre, single-blind randomised controlled trial designed to evaluate if an IVC and lung ultrasound-guided healthcare strategy is superior to standard care to reduce subclinical congestion at discharge. Fifty-eight patients with AHF will be randomised using a block randomisation programme that will assign to either lung and IVC ultrasound-guided decongestion therapy (‘intervention group’) or clinical-guided decongestion therapy (‘control group’), using a quantitative protocol and will be classified in three groups according to the level of congestion observed: none or mild, moderate or severe. The treating physicians will know the result of the test and the subsequent adjustment of treatment in response to those findings guided by a customised therapeutic algorithm. The primary endpoint is the presence of more than five B-lines and/or an increase in the diameter of the IVC, with and without collapsibility. The secondary endpoints are the composite of readmission for HF, unplanned visit for worsening HF or death at 90 days, variation of pro-B-type natriuretic peptide at discharge, length of hospital stay and diuretic dose at 90 days. Analyses will be conducted as between-group by intention to treat.

**Ethics and dissemination:**

Ethical approval was obtained from the Institutional Review Board and registered in the PRIISA.BA platform of the Ministry of Health of the City of Buenos Aires.

**Trial registration number:**

NCT04549701.

**WHAT IS ALREADY KNOWN ON THIS TOPIC**

- Remaining clinical congestion at discharge is a strong predictor of mortality and readmissions in patients hospitalised for acute heart failure (AHF). However, the optimal assessment of decongestion and how to guide treatment are not clearly defined.

**WHAT THIS STUDY ADDS**

- The inferior vena CAVA and Lung UltraSound-guided therapy in Acute Heart Failure (CAVAL US-AHF) is a single-centre, single-blind randomised controlled trial. It will provide evidence on the usefulness of this innovative ultrasound protocol that evaluates both right-sided congestion through the evaluation of the inferior vena cava and left-sided congestion through lung ultrasound to guide pulmonary decongestion in patients hospitalised for AHF.

**HOW THIS STUDY MIGHT AFFECT RESEARCH, PRACTICE OR POLICY**

- CAVAL US-AHF could provide evidence on the use of a simple, non-invasive technique to guide treatment during hospitalisation for AHF with the goal of achieving clinical and subclinical decongestion to potentially decrease the risk of events after discharge.

## Introduction

Despite the important therapeutic advances, the prevalence of heart failure (HF) is increasing^1^ and is still a major healthcare issue due to its high morbidity and mortality.^2^ Approximately 25%–30% of patients hospitalised for acute HF (AHF) are readmitted within 90 days after discharge, and 50% within 6 months.^3–5^ The post-discharge period, known as the ‘vulnerable phase’,^6^ is associated with high risk of unfavourable outcomes.^7 8^ Readmission of these patients does not only increase costs but is also a signal that current approaches to HF management are not optimal. In fact, the greatest threat to patients with AHF is the risk of readmission due to persistent congestion.^9–11^

Remaining clinical congestion at discharge is a strong predictor of mortality risk^12 13^; therefore, its assessment prior to hospital discharge remains a crucial opportunity to treat patients who have not yet reached the optimal euvolemia. However, the optimal evaluation of decongestion in AHF is not clearly defined in the guidelines,^14 15^ which may be partly explained by the lack of effective measurement methods.^10 16^ Monitoring patient fluid status is a dynamic and challenging process with a broad spectrum of clinical presentations and parameters to consider. Physicians are faced with the daily task of making critical decisions with a handful of tools and a significant gap in evidence as above mentioned.^17^

Evaluation and monitoring of fluid excess status in patients admitted for acute decompensated HF are currently based on clinical history, physical examination, chest X-ray and natriuretic peptides.^10^ However, all these elements have an inherent substantial interobserver variability and may be non-specific,^18 19^ and plasma levels of biomarkers have a limited capacity to assess quantitatively the extent of fluid retention.^20^

Lung ultrasound (LUS) has been introduced in the evaluation of pulmonary congestion.^21^ The number of B-lines is a reliable marker of the presence of extravascular lung water and has allowed the identification of patients with HF with worse prognosis. Several publications have demonstrated the usefulness of B-lines in the outpatient follow-up of patients with chronic HF to reduce the number of hospitalisation rates due to AHF.^22–24^ In patients hospitalised for AHF, residual pulmonary congestion assessed by LUS was a strong predictor of short-term mortality and rehospitalisation.^25–28^ Recently, a study analysed the prognostic capacity of the presence of B-lines in patients in whom their treating physicians considered them properly lung decongested. Up to 40% of patients, considered decongested according to pulmonary auscultation, present subclinical congestion at hospital discharge which implies a worse prognosis at 6-month follow-up.^28^ This congestion can be detected by LUS.

Measurement of the inferior vena cava (IVC) diameter by ultrasound is a simple method to estimate right atrial pressure and is associated with changes in pulmonary capillary pressure.^29^ Increased diameter and collapsibility of the IVC in the outpatient setting predict a higher risk of hospitalisation and mortality due to AHF.^30 31^ In patients hospitalised for AHF, therapy guided by IVC diameter is associated with a reduction in 30-day readmission^32^ and mortality rates.^33^

In a small recent pilot study, a protocol combining focused echocardiographic evaluation of cardiac filling pressures, and IVC index with LUS resulted safe and reliable for guiding treatment in hospitalised patients with AHF.^34^

To date, no randomised trials have investigated the usefulness of the evaluation of right-sided congestion through the IVC and left-sided congestion through LUS to guide therapy in hospitalised patients with AHF, with the aim of improving the decongestion at discharge.

Inferior vena CAVA and Lung UltraSound-guided therapy for the reduction of clinical events in Acute Heart Failure (CAVAL US-AHF) pilot trial has been designed to evaluate if IVC and LUS-guided therapy is superior to standard care to reduce subclinical congestion at discharge, and secondarily, to explore whether it reduces clinical events at 90 days. This report describes the rationale and study design of this trial.

## Methods

**Figure 1:**
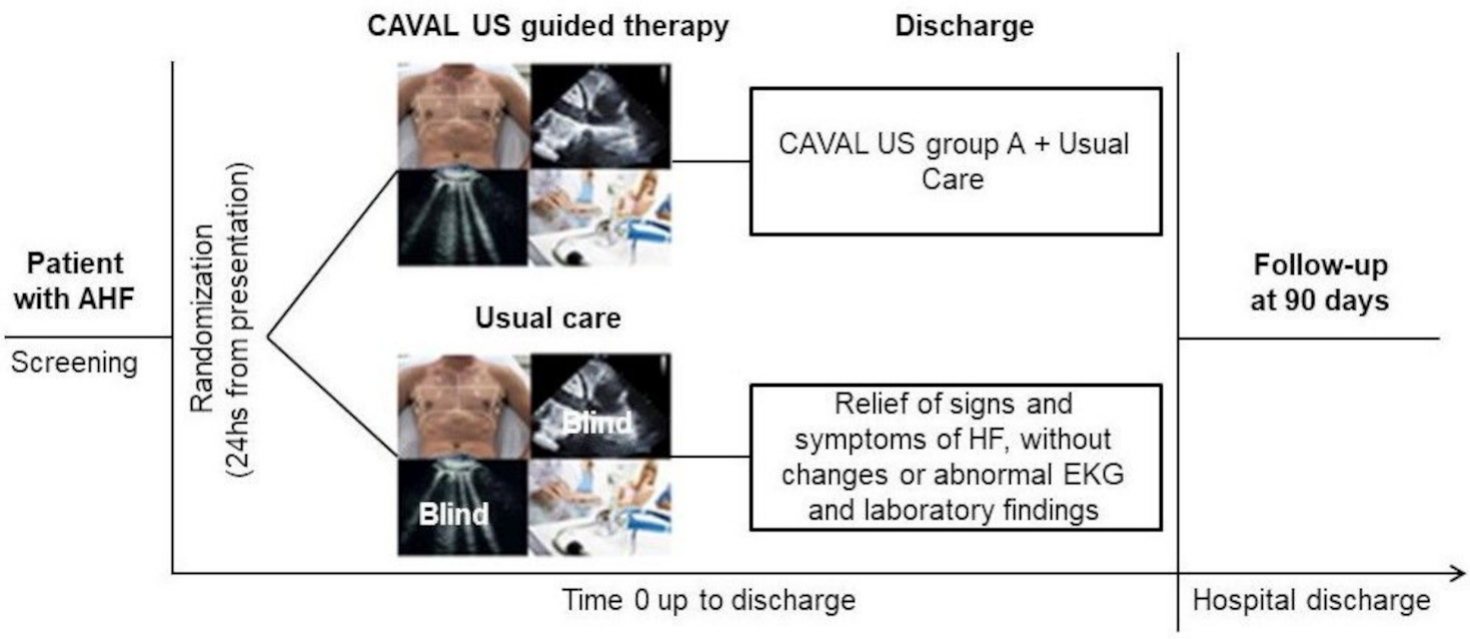
Trial diagram. AHF, acute HF; CAVAL US, inferior vena cava and lung ultrasound; HF, heart failure.

We designed a single-centre, single-blinded, randomised controlled clinical trial (NCT04549701). The flow diagram of the trial is shown in figure 1.

### Participants and eligibility

Patients admitted with AHF in a private cardiovascular centre in Buenos Aires, Argentina. Eligibility criteria for participants are described in box 1.

Box 1 **Eligibility criteria**

**Inclusion criteria (all of them)**

- Hospitalisation of 24 hours or more for decompensated heart failure (HF) defined as new onset of symptoms or worsening of previous symptoms (including orthopnoea, progression to functional class New York Heart Association III–IV, bendopnoea or fatigue) or signs of volume overload.

- Jugular venous distension, hepatojugular reflux, lower extremity oedema or signs of pulmonary congestion.
- Chest X-ray with signs suggestive of pulmonary congestion.

- Elevated pro-B-type natriuretic peptide levels of 450 pg/mL, 900 pg/mL and 1800 pg/mL for ages <50 years, 50–75 years and >75 years, respectively, within 24 hours of admission.^53^ ^54^

- Sufficient ultrasound visualisation to assess inferior vena cava and lungs.

**Exclusion criteria (any of them)**

- Not willing to participate.
- Life expectancy of less than 6 months.
- Uninterpretable lung or inferior vena cava ultrasound.
- Transfer to another hospital before hospital discharge.
- Systolic blood pressure <90 mm Hg.
- Chronic kidney disease (creatinine clearance <30 mL/min calculated with the Modification of Diet in Renal Disease (MDRD) study equation or haemodialysis).
- Requirement for invasive or non-invasive ventilator support.
- Pregnancy.
- Low cardiac output syndrome/cardiogenic shock.
- Death during index hospitalisation.
- Acute coronary syndrome, myocardial revascularisation or heart valve replacement within the previous 3 months.
- Being on heart transplant waiting list.
- Cardiac resynchronisation therapy device implanted within the previous 3 months.
- Severe tricuspid valve regurgitation.
- HF secondary to causes amenable to invasive correction: cardiac surgery, percutaneous interventions or pacemaker implantation.
- HF secondary to significant arrhythmias (advanced atrioventricular block or sinus arrest, sustained ventricular tachycardia or any sustained arrhythmia other than atrial fibrillation causing haemodynamic instability according to the discretion of the treating physician).
- HF secondary to severe systemic infection.
- Severe psychiatric illness.
- Palliative care.
- SARS-CoV-2 infection.

### Randomisation

Patients will be randomised using a block randomisation programme that will assign to either lung and IVC ultrasound-guided decongestion therapy (‘intervention group’) or clinical-guided decongestion therapy (‘control group’). Randomisation will be stratified by age (<70 or >70 years) and by left ventricular function (<50% or >50%). Randomisation will be performed within 24 hours after hospital admission using the REDCap randomisation module with a 1:1 allocation scheme.

### Study intervention

Our hospital practice is based on clinical guidelines,^15^ and all the patients admitted for HF will be evaluated and treated by the same medical team, composed of physicians specialised in cardiovascular diseases.

**Figure 2:**
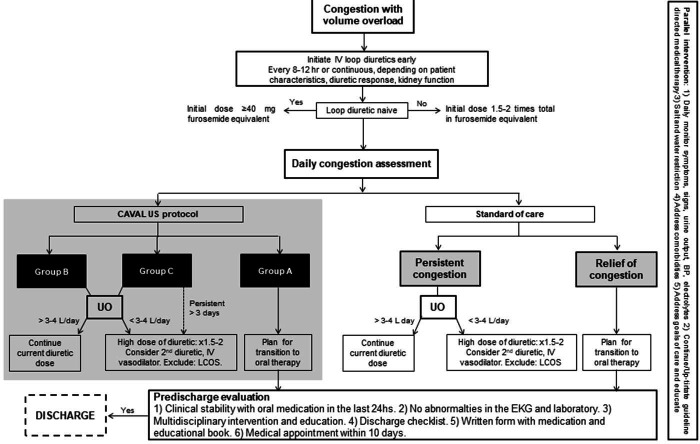
Therapeutic algorithm. Adapted from: Mullens *et al* 35 and Hollenberg *et al*.^36^ BP, blood pressure; CAVAL US, inferior vena cava and lung ultrasound; IV, intravenous; LCOS, low cardiac output syndrome; UO, urinary output.

The diuretic treatment algorithms proposed by the Heart Failure Association of the European Society of Cardiology^35^ and the American College of Cardiology^36^ were adapted, and the CAVAL US protocol was added as part of the daily assessment of congestion (figure 2).

All the patients will undergo lung and IVC ultrasound, independently of the group assigned during randomisation. Furthermore, an echocardiogram will be performed within 48 hours from admission according to the recommendations,^15^ as part of our standard of care.

A checklist will be made prior to hospital discharge to assess compliance with guideline-based treatment, vaccination, counselling and medical appointment after discharge. All patients will receive education by an HF nurse, who will provide educational material and drug treatment indications in a written grid.

Patients will be randomised into two arms and will be blinded to the assigned arm:

1. Control group: the treating medical team will be blinded to the lung and IVC ultrasound results. Patients will receive the standard of care of our centre and titration of diuretics will be based on standard practices (physical examination, symptoms and results of laboratory tests). The therapeutic goal (see figure 2) will be to discharge patients with relief of signs and symptoms of congestive HF, without ECG changes or abnormal laboratory findings that contraindicate hospital discharge. Circulating biomarkers of congestion are not part of the standard of care.
2. Intervention group: the treating medical team will be unblinded to the lung and IVC ultrasound results. Patients will receive treatment for decongestion according to the results of the intervention plus the standard care (figure 2). The therapeutic objectives will be similar to the control group, with relief of the signs and symptoms of HF and additionally without congestion or mild residual congestion in the ultrasound test. At the discretion of the treating physician, the patient may be discharged when he or she shows mild to moderate signs of congestion in improvement with oral diuretics for more than 24 hours with adequate diuretic response.

Both groups will be scheduled to follow-up visits in our ambulatory clinic 7–10 days after discharge, and at least once per month.

### Lung and IVC ultrasound protocol

All the patients will undergo lung and IVC ultrasound using a Philips Lumify hand-held ultrasound device. The ultrasound will be performed by physicians specialised in lung and cardiovascular ultrasound and will be quantified off-line in an echocardiography laboratory made up of three independent physicians who will be blinded to the study group, patient information and interpretation of the other expert reviewer.

**Figure 3:**
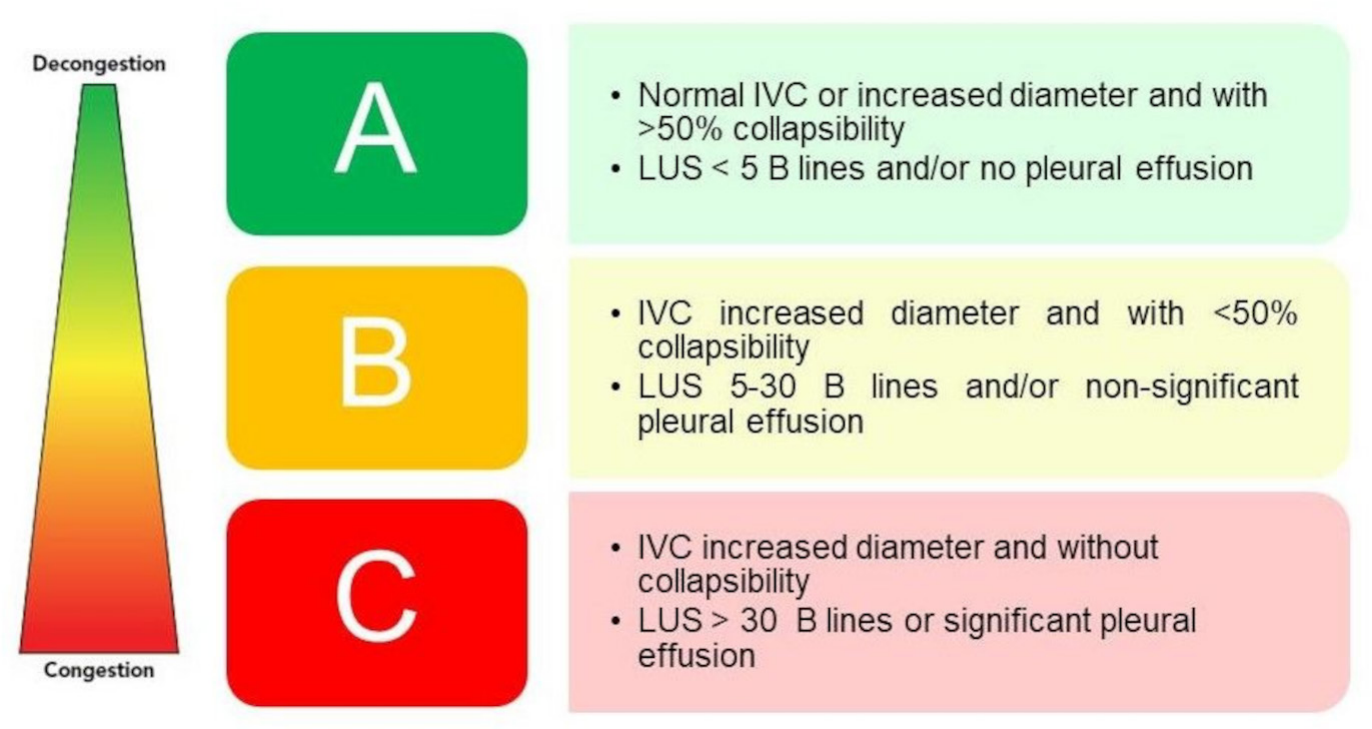
CAVAL US groups. IVC, inferior vena cava; LUS, lung ultrasound.

The results of the ultrasound will be reported in three categories (figure 3):

Group A—no congestion or mild residual congestion.

Group B—signs of right-sided or left-sided congestion or both.

Group C—severe signs of right-sided or left-sided congestion and worse prognosis.^2 25 32 33 37^ The cut-off value of ≥5 B-lines was chosen as sign of persistent congestion, since it was found to be a risk factor for the occurrence of the primary endpoint of rehospitalisation, unexpected visit for worsening HF or death at 6-month follow-up.^28^ The presence of >30 B-lines is considered severe congestion as it proved to be a strong predictor of hospitalisation for HF or mortality at 90 days (HR 5.66, 95% CI 1.74 to 18.39).^25^

### Technique and quantification

IVC assessment: the IVC is assessed as recommended in the current echocardiography guidelines. Cardiac transducer of 3–7 MHz, in the cardiac preset, depth of 10 cm. The patient is kept in a supine position with the least elevation of the upper body possible (<20°) The IVC is scanned in its long axis from the subcostal view. The measurement of the IVC diameter is performed just proximal (1.5 cm) to the entrance of the hepatic veins at end expiration (IVCmax) and at inspiration (IVCmin). The IVC collapsibility index is derived from these parameters as percentage collapse of the maximal IVC diameter as follows: (IVCmax−IVCmin)/IVCmax×100. An IVC diameter <21 cm with collapsibility >50% is normal, while IVC is dilated when its diameter is >21 mm and collapsibility <50%.^38^

LUS: the procedure will be performed using a portable ultrasound device. The eight-zone method will be used to evaluate eight chest zones (four zones in each hemithorax) according to international recommendations.^39^ Dividing lines include the sternum medially, the anterior axillary line dividing medial and lateral zones, and the posterior axillary line laterally. The third intercostal space divides the superior and inferior zones.

The probe will be positioned perpendicular to the ribs, with a scanning depth of 16 cm and the patient in semirecumbent position. Gain will be adjusted to each patient so that the shadows of the ribs are black and the pleural line with lung sliding is seen clearly. A 6-second video clip of each zone will be recorded. B-lines are defined as comet-tail, vertical artefacts arising from the pleural line, moving in synchrony with lung sliding (when present), well defined and laser like, extending to the bottom of the screen without fading. The highest number of B-lines (vertical lines arising from the pleural line) visualised in a single intercostal space will be recorded for each zone. The white lung pattern is counted as 10 B-lines, and fused B-lines are counted as the percentage of the rib space filled with confluent B-lines divided by 10 and added to any other B-lines noted in the space at that instant.^39^ A positive region is defined by the presence of three or more B-lines in a longitudinal plane between two ribs.^21^ The presence of pleural effusion will also be evaluated, and its size will be categorised offline on 6-second clips using a semiquantitative score called pleural effusion (PEF) score, ranging from 0 to 4 points for each hemithorax (box 2) with a total score ranging from 0 to 8. Significant pleural effusion will be defined as a PEF score of 5–8 and non-significant pleural effusion when PEF score is less than 5.^40^

Box 2 **Definition of the pleural effusion (PEF) score for each hemithorax.^40^**

- 4—pleural effusion occupies more than 50% of the basal pleural cavity visible in the standardised imaging plane.
- 3—clear separation between diaphragm and lung base at any point during the respiratory cycle.
- 2—pleural effusion extends over the costophrenic angle without a clear separation of the lung base from the diaphragm.
- 1—pleural effusion is only visible in the costophrenic angle.
- 0—pleural effusion is not visible.

The box has been reproduced with permission from Lindner *et al*.^40^

Before the trial began, cardiologists and ultrasound specialists attended a workshop for specific technical training to standardise the way the examination is conducted, interpreted and reported. The workshop lasted 24 hours (18 hours of theory and 6 hours of practice).

### Study endpoints and follow-up

The primary endpoint is the presence of subclinical congestion at discharge, defined as the presence of more than five B-lines and/or an increase in the diameter of the IVC, with and without collapsibility.

The secondary endpoint is the composite of readmission for HF, unplanned visit for worsening HF or death at 90 days. Additionally, we will assess other secondary endpoints such as the variation of pro-B-type natriuretic peptide at discharge, length of hospital stay, total number of HF hospital admissions and diuretic dose at 90 days (box 3). AHF hospitalisation was defined as unscheduled urgent hospital visit and hospital stay >24 hours, requiring intravenous HF therapies (diuretics, vasodilators, inotropes). Urgent AHF visits were defined as unscheduled visit to the emergency department resulting in increased dose of oral/intravenous therapy, stay <24 hours.

Box 3 **Secondary endpoints**

- Composite outcome: readmission for heart failure (HF), unplanned visit for worsening HF or death at 90 days.
- All-cause mortality at 90 days.
- Duration of index hospital stay.
- Reduced total readmissions (first and recurrent) for HF at 90 days.
- Increased requirement for diuretics: patients who required a higher dose of furosemide compared with the dose indicated on hospital discharge.
- Pro-B-type natriuretic peptide (NT-proBNP) at discharge.
- % change of NT-proBNP (admission–discharge).

The safety endpoints during the index hospitalisation include:

- Hypotension (systolic blood pressure <90 mm Hg).
- Requirement of vasoactive drugs (inotropic/pressor agents).
- Worsening renal function leading to a creatinine increase of ≥50% or >0.3 mg/dL on any of the blood tests performed during the index hospitalisation.
- Hypokalaemia <3.5 mmol/L or hyperkalaemia >5 mmol/L.

At 90 days after randomisation, two independent physicians, blinded to the assigned group, will adjudicate events by telephone contact. In case of disagreement, a third independent physician will evaluate the case.

### Subgroup analysis

The primary endpoint of subclinical congestion at discharge will be evaluated by baseline left ventricular ejection fraction (<50% and >50% or greater), sex, comorbidities, cardiomyopathy aetiology, and age >70 years or <70 years.

### Allocation concealment and masking procedures

Lung and IVC ultrasound will be performed to all the patients, and patients will be blinded to the group assigned. The treating medical team will be blind to the results of the ultrasound of the control group. The independent clinicians adjudicating 90-day events will not participate in patient follow-up and will be blind to the assigned group.

### Statistical analysis plan

According to our preliminary pilot data derived from a sample of 20 patients during the new ultrasound tool proof trial, 70% had >5 B-lines and/or dilated IVC at discharge with the usual care strategy at our hospital. Therefore, to reach a relative risk reduction of 50% (ie, 70% in usual care and 35% in the CAVAL US-guided strategy), setting a power of 80% and a two-sided type I error rate of 5%, we will need 29 patients in each arm to accomplish our hypothesis.

Analysis will be performed by intention to treat. Continuous variables will be expressed as mean and SD, or median and IQR, according to their distribution. Normality of distribution of variables will be assessed using the Kolmogorov-Smirnov test or the Shapiro-Wilk test, according to the sample size. Continuous variables will be compared using the Student’s t-test or the Mann-Whitney U test, as applicable. Categorical variables will be presented as numbers and percentages. The Χ^2^ test or Fisher’s exact test will be used to compare proportions. No imputation will be made for missing data.

The Kaplan-Meier method will be used to analyse time-to-first event data. The differences in time-to-event distributions will be evaluated using the log-rank test. Univariate HRs with associated 95% CIs will be estimated for the composite primary endpoint and secondary endpoints and derived from the Cox proportional-hazards model; however, multivariate Cox regression analysis will only be used if there are important prognostic factors or patients’ baseline characteristics exhibiting significant imbalance between the two groups established by randomisation. In all cases, the alpha error will be set at 5% to establish statistical significance. All the statistical calculations will be performed using SPSS V.24 software package.

### Confidentiality

The investigators and the Institutional Review Board of Instituto Cardiovascular de Buenos Aires will implement measures to protect the confidentiality of all the information according to the Argentine personal data protection law 25.326. These records will be kept confidential. The participants will only be identified by using numbers or letters as identification code. The identity of the participants will not be revealed if the results of the study are published.

## Discussion

Residual congestion at hospital discharge is one of the major factors contributing to readmission for HF,^13 41^ even in patients without clinical signs of congestion,^42^ possibly due to the persistence of subclinical overload.

Since the advent of LUS, we have gained a broader notion of the sequence of events leading to pulmonary oedema starting with increase in left ventricle end-diastolic pressure that translates into an increased pulmonary capillary wedge pressure. This phenomenon is called haemodynamic congestion and is the initial cause to break the Starling’s equilibrium in the alveolar–capillary barrier, resulting in lung fluid overload.^43^ The intermediate event between haemodynamic and clinical pulmonary congestion is subclinical pulmonary congestion, detectable by LUS as multiple B-lines.^44^ Clinical congestion becomes evident hours, days or weeks later.

Detection, dynamic monitoring, and management of clinical and subclinical congestion could help improve prognosis, especially during the vulnerable period following hospitalisation for AHF. The assessment of pulmonary congestion by LUS and of systemic congestion in the IVC with a standardised quantitative approach could represent a valuable and novel tool to guide the management of these patients.

Among the limitations of our proposed study, the fact that it will be performed in a high-complexity single centre dedicated to cardiovascular diseases could hamper the generalisation of the results. Nevertheless, the broad inclusion criteria established and the simplicity of the CAVAL US protocol indicate that this ultrasound-guided treatment strategy could be applied in centres of less complexity. Second, the treating physicians responsible for treatment decisions are experienced cardiologists working in a multidisciplinary programme composed by a specialised nurse. This could reduce the number of events in both groups, reducing not only the power of the trial, but also the potential benefits of ultrasound-guided therapy by improving the evaluation in the control group due to the high expertise of the professionals. Nevertheless, we have proposed an algorithm for standardised care that will allow reproducibility of treatment in other centres.

The therapeutic algorithm was adapted from the two most recent consensus statements published by the Heart Failure Association of the European Society of Cardiology^35^ and the American College of Cardiology,^36^ which have not been yet validated but were based on experts’ opinion. Further, in the suggested daily evaluation of congestion, in the CAVAL US-guided therapy arm, the clinical assessment is complemented by ultrasound findings to detect subclinical abnormalities.

The treating medical team will be blinded to the results of the ultrasound of the control group and the patients will be blinded to the arm assigned as a consequence of the nature of the study. Furthermore, while there is no way to blind the patient’s clinical status to the team performing the ultrasound examinations, all the ultrasounds will be interpreted and reported by a Core Lab, made up of two expert sonographers, blinded to the clinical characteristics of the patient.

The evaluation of congestion is extremely difficult, especially when a binary definition, such as present or absent, is required. Clinical scores combining several clinical indicators have proven to better assess the level of congestion than any independent indicator.^44^ Recognising that there are many indicators of pulmonary congestion, we focused on a comprehensive assessment of clinical congestion by means of the EVEREST risk score.^45^

Lung and IVC ultrasound are simple techniques; one morning hands-on experience or even a standardised internet-based module of 2 hours is sufficient to achieve excellent reproducibility in identification and quantification of B-lines, even among LUS-naive sonographers.^46^ The operators will attend a theory course and two workshops to practise the technique, standardise the way the examination is conducted and improve correlation between those who interpret the images.

To date, neither the international evidence-based recommendations nor the expert consensus document for quantification of pulmonary congestion by LUS in HF has recommended how to quantify B-lines in each intercostal space.^39 46^ Therefore, the method of counting B-lines noted in the intercostal space at any instant was chosen, since it is technically simpler to perform and more reliable than the other methods previously reported.^47^ In addition, and opposed to previous publications,^22 24^ longer LUS clips will be recorded in each lung zone since the number of B-lines that can be detected with longer clips in patients with HF is greater.^48 49^

It is worth mentioning that several approaches have been proposed to estimate the volume and severity of pleural effusion.^50–52^ However, their value in patients with AHF may be limited. This is because some of these techniques require several measurements that are time-consuming, while others can only be applied for moderate to large effusions, as they were primarily developed to predict safety of thoracentesis. A semiquantitative score for pleural effusion in patients with AHF has been recently suggested.^40^

Finally, lung and IVC ultrasound is a simple, non-invasive technique that does not require the use of Doppler or complex methods of quantification, is inexpensive and can be performed with a hand-held device that can be easily sanitised. This is an extremely important aspect considering that the trial will be conducted during the COVID-19 pandemic.

## Conclusion

The CAVAL US-AHF clinical trial will provide evidence on the usefulness of this innovative ultrasound protocol that evaluates both right-sided congestion through the evaluation of the IVC and left-sided congestion through LUS to guide pulmonary decongestion in patients hospitalised for AHF.

## Data Availability

Data are available upon reasonable request.
